# Analysis of the Transmissibility Change of 2019-Novel Coronavirus Pneumonia and Its Potential Factors in China from 2019 to 2020

**DOI:** 10.1155/2020/3842470

**Published:** 2020-05-18

**Authors:** Yu Zhao, Ruonan Wang, Jiangping Li, Yuhong Zhang, Huifang Yang, Yi Zhao

**Affiliations:** School of Public Health and Management, Ningxia Medical University, Yinchuan Ningxia, China 750004

## Abstract

**Background:**

Recently, a large-scale novel coronavirus pneumonia (NCP) outbreak swept China. As of Feb. 9, 2020, a total of 40,260 patients have been diagnosed with NCP, and 23,589 patients were suspected to have infected by the 2019 novel coronavirus (COVID-19), which puts forward a great challenge for public health and clinical treatment in China. Until now, we are in the high-incidence season of NCP. Thus, the analysis of the transmissibility change of NCP and its potential factors may provide a reliable reference for establishing effective prevention and control strategies.

**Method:**

By means of the method of calculating the instantaneous basic reproduction number *R*_0*t*_ proposed by Cori et al. (2013), we use *R*_0*t*_ to describe the transmissibility change of COVID-19 in China, 2019-2020. In addition, the Baidu Index (BDI) and Baidu Migration Scale (BMS) were selected to measure the public awareness and the effect of Wuhan lockdown (restricted persons in Wuhan outflow from the epidemic area) strategy, respectively. The Granger causality test (GCT) was carried out to explore the association between public awareness, the effect of the Wuhan lockdown strategy, and the transmissibility of COVID-19.

**Results:**

The estimated averaged basic reproduction number of NCP in China was 3.44 with 95% CI (2.87, 4.0) during Dec. 8, 2019, to Feb. 9, 2020. The instantaneous basic reproduction numbers (*R*_0*t*_) have two waves and reaching peaks on Jan. 8 and Jan. 27, respectively. After reaching a peak on Jan. 27, *R*_0*t*_ showed a continuous decline trend. On Feb. 9, *R*_0*t*_ has fallen to 1.68 (95% CI: 1.66, 1.7), but it is still larger than 1. We find a significantly negative association between public awareness and the transmissibility change of COVID-19, with one unit increase in cumulative BDI leading to a decrease of 0.0295% (95% CI: 0.0077, 0.051) *R*_0*t*_. We also find a significantly negative association between the effect of the Wuhan lockdown strategy and the transmissibility change of COVID-19, and a one unit decrease in BMS may lead to a drop of 2.7% (95% CI: 0.382, 4.97) *R*_0*t*_.

**Conclusion:**

The current prevention and control measures have effectively reduced the transmissibility of COVID-19; however, *R*_0*t*_ is still larger than the threshold 1. The results show that the government adopting the Wuhan lockdown strategy plays an important role in restricting the potential infected persons in Wuhan outflow from the epidemic area and avoiding a nationwide spread by quickly controlling the potential infection in Wuhan. Meanwhile, since Jan. 18, 2020, the people successively accessed COVID-19-related information via the Internet, which may help to effectively implement the government's prevention and control strategy and contribute to reducing the transmissibility of NCP. Therefore, ongoing travel restriction and public health awareness remain essential to provide a foundation for controlling the outbreak of COVID-19.

## 1. Introduction

Recently, a large-scale novel coronavirus pneumonia (NCP) outbreak swept China, and NCP cases have also been reported in several countries internationally, such as Singapore, Republic of Korea, Japan, Italy, and Malaysia [1, 2]. As of Feb. 9, 2020, a total of 40,171 patients have been diagnosed with NCP in China; 23,589 patients were suspected to have infected by COVID-19 now, and a total of 399,487 close contacts were traced [3], which puts forward a great challenge for the public health and clinical treatments in China. Until now, the epidemic of COVID-19 still shows a high number of new confirmed cases and suspected infections per day. Thus, estimating the change of transmissibility of COVID-19 may quantitatively assess the severity of outbreak and provide evidence for more effective public health decisions based on empirical data, which can maximally control the outbreak of NCP and reduce socioeconomic losses.

**Figure 1:**
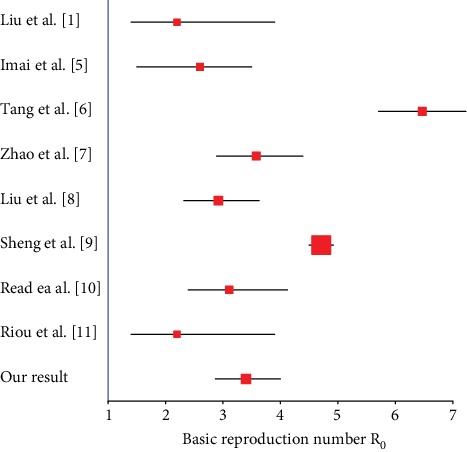
The summary forest plot of the basic reproduction number (*R*_0_) of COVID-19.

One of the most important parameters in epidemiological dynamics is the basic reproduction number (*R*_0_), which can be thought of as the expected number of cases directly generated by one case in a population where all individuals are susceptible to infection [4]. Thus, the basic reproduction number can represent the transmissibility of an infectious disease. Since the outbreak of COVID-19, there are many researchers who established some mathematical models to estimate the basic reproduction number *R*_0_ of COVID-19. For example, Imai et al. [5] estimated an averaged *R*_0_ of 2.6 with an uncertainty range 1.5-3.5 up to 18th January 2020, based on an analysis combining their past estimates of the size of the outbreak in Wuhan with computational modelling of potential epidemic trajectories, and predicted that the control measures need to block well over 60% of transmission to be effective in controlling the outbreak. On the basis of the information of 425 patients with confirmed NCP, Li et al. [1] estimated the basic reproduction number to be 2.2 (95% CI: 1.4-3.9). Tang et al. [6] obtained the estimation results based on the likelihood and model analysis, which reveal that the basic reproduction number may be as high as 6.47 (95% CI: 5.71-7.23). Accounting for the impact of variations in disease reporting rate, Zhao et al. [7] estimated that the mean *R*_0_ ranges from 2.24 (95% CI: 1.96-2.55) to 3.58 (95% CI: 2.89-4.39) associated with an 8-fold to 2-fold increase in the reporting rate. Exponential growth (EG) and maximum likelihood estimation (ML) were applied to estimate the reproductive number of 2.92 (95% CI: 2.32-3.63) [8]. Sheng et al. [9] estimated the basic reproduction number *R*_0_ to be 4.71 (95% CI: 4.50-4.92) based on the dynamical model, and the effective reproduction number has dropped to 2.08 (1.99-2.18) as of Jan. 22, 2020. More related results [10, 11] with respect to the *R*_0_ of COVID-19 recently are displayed in a summary forest plot (see Figure1 for more details).

Shortly after COVID-19 confirmed cases swept China, all 31 provincial-level regions in China activated top-level prevention and control measures. The implementation of these control strategies depends on the public's response, such as raising public health awareness, and on Jan. 23, 2020, Wuhan was locked down, which restricted the persons in Wuhan outflow from the epicenter area. These strategies may be related to the transmissibility change of COVID-19, and how to quantitatively measure the effect of public awareness and the Wuhan lockdown strategy on the transmissibility change of COVID-19 is one of the key issues. As far as we know, there are rare results with respect to this.

Therefore, to assess the transmission pattern of COVID-19 and potential factors, in this paper, we estimated the transmissibility of COVID-19 recently and explored the relationship between instantaneous basic reproduction number (*R*_0*t*_), public awareness, and the effect of the Wuhan lockdown strategy.

## 2. Materials and Methods

The daily confirmed cases are obtained from the National Health Commission of the People's Republic of China [3] and the Health Commission of Hubei Province [12]. The daily confirmed cases from Dec. 8, 2019, to Jan. 20, 2020, were obtained from Li et al. [1].

Assessing the transmissibility change of infectious diseases during the epidemic period is the foundations for designing and adjusting the public health response. Transmissibility can be measured by the basic reproduction number, which can also reflect the effectiveness of interventions and the intensity of control efforts. Cori et al. [13] proposed a tool for estimating the instantaneous basic reproduction number *R*_0*t*_ from incidence time series and serial interval (SI, the time between the onset of symptoms in a primary case and the onset of symptoms of secondary cases). The model for estimating *R*_0*t*_ at *t* day is given as follows:

(1) $$\begin{array}{c} R_{0t}=\frac{I_{t}}{\sum_{k=0}^{n}\omega \left(k\right)I_{t-k}}, \end{array}$$

where *I*_*t*_ is the confirmed case of COVID-19 at *t* day, *ω*(*k*) is the weighted function determined by the distribution of SI of COVID-19, ∑_*k*=0_^*n*^*ω*(*k*)*I*_*t*−*k*_ is the sum of infection incidence up to the time step *t* − 1, and *n* denotes the maximum of the window size. Li et al. [1] estimated the serial time (SI) of COVID-19. The mean of SI is 7.5 days, and the standard deviation of SI is 3.5 days.

The Baidu Index [14] (BDI) is based on the search volume of the netizen in Baidu as the database, and keywords are used as statistical objects. The weighted sum of the search frequency of each keyword in the Baidu web search was calculated as BDI (more in Appendix A). We collected the BDI data in the Chinese term “new coronavirus,” and the data can be found in an online website (the reason why we selected this keyword is given in Appendix B).

In addition, the Baidu Migration Scale (BMS) [15] reflects the size of the population moving in or out in one place in China (more details can be seen in Appendix A). We selected Wuhan city as the emigration destination in BMS and collected the daily BMS from Jan. 1, 2020, to Feb. 9, 2020. We consider the BDS as a proxy of the effect of the Wuhan lockdown strategy.

Motivated by Zhao et al. [16], we used the following univariate regression to quantitatively explore the effect of public awareness and the Wuhan lockdown strategy on the transmissibility changes of COVID-19:

(2) $$\begin{array}{c} E\left(\ln R_{0t}\right)=\beta_{1}{\text{factor}}_{t}+\beta_{0}+\varepsilon_{t}, \end{array}$$

where *E*() denotes the expectation of the response variable, factor_*t*_ is the potential factor at *t* day (such as BDI or BMS), *ε*_*t*_ is a random error subjected to the normal distribution with 0 mean and constant variation, *β*_1_ is the regression coefficient, and exp(*β*_1_) − 1 is the transmissibility change percentage. To avoid spurious regression, we also performed the Granger causality test (GCT) [17, 18] to analyze the causality between the transmissibility change and BDI (or BMS). The order of GCTs was determined by the partial autocorrelation functions (PACF). GCT can often be used to examine the possible causal relationship between two time series, that is, the past behavior of one sequence may affect the current behavior of the other. The GCTs are significant, which means that there may be a causal relationship between public health awareness, the Wuhan lockdown strategy, and the instantaneous basic reproduction number *R*_0*t*_ of COVID-19.

Analysis of GCT was performed with the use of STATA with the package “gcause,” and other analyses were performed with the use of the MATLAB software (MathWorks, Version 2012a) and R software (R Project for Statistical Computing, Version 3.6.2).

## 3. Results

**Figure 2:**
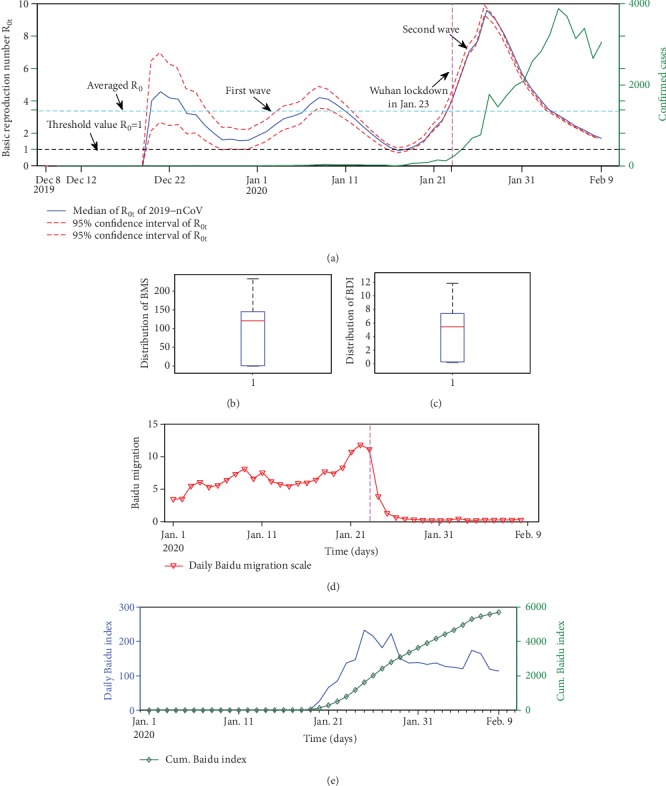
(a) The instantaneous basic reproduction number *R*_0*t*_ and 95% confidence interval of NCP in China 2019 (solid blue line is the median of *R*_0*t*_, and red dotted lines are the 95% confidence interval of *R*_0*t*_) and the daily confirmed cases time series of NCP (solid green line). (b) The boxplot of the Baidu Migration Scale Index. (c) The boxplot of the Baidu Index. (d) The time series of daily Baidu Migration scale. (e) The time series of daily Baidu Index.

We estimated the instantaneous basic reproduction number *R*_0*t*_ as shown in Figure 2. From Dec. 8, 2019, to Feb. 9, 2020, the averaged basic reproduction number *R*_0_ of NCP in mainland China was 3.51 (95% CI: 2.91-4.09), which is consistent with the result of Zhao et al. [7]. The instantaneous basic reproduction numbers have two waves and reaching peaks on Jan. 8 and Jan. 27, respectively. After Jan. 27, 2020, *R*_0*t*_ showed a continuous decline trend and dropped to 1.68 (95% CI: 1.66, 1.7) on Feb. 9, 2020.

We find negative association between the instantaneous basic reproduction number *R*_0*t*_ and cumulative BDI (lag time from 1 to 12 days) and negative relationship between instantaneous basic reproduction number *R*_0*t*_ and BMS (see Table 1). It implies that the cumulative BDI and BMS are the Granger causes for *R*_0*t*_. More precisely, the optimal goodness of fit appeared at lags 12 days for cumulative BDI with an R-square of 0.285. Fitting results showed an increase of one unit of cumulative BDI (10 thousand terms of search with respect to the information of “novel coronavirus” in the Baidu search engine), leading to a decrease of 0.0295% (95% CI: 0.0077, 0.051) *R*_0*t*_. The decline in the 0.0295% of transmissibility of COVID-19 may be due to the improvement of public self-protection awareness by searching for relevant information via the Internet.

From Figure 2(d), we observed that the outflow rate of Wuhan suddenly dropped to a very lower value after the Wuhan lockdown strategy was carried out on Jan. 23, 2020 (BMS decreased from 11.4 on Jan. 23 to 1.3 on Jan. 25). We also find a significantly negative association between the effect of Wuhan lockdown and the transmissibility change of COVID-19 (Table 1), and a one unit a decrease in BMS may lead to a drop of 2.7% (95% CI: 0.382, 4.97) *R*_0*t*_. The R-square value is 0.137 for *R*_0*t*_ versus BMS. Thus, the Wuhan lockdown strategy may contribute to 2.7% of the transmissibility change of COVID-19.

## 4. Conclusion

The outbreak of NCP recently seriously affected the people's health and socioeconomic development in China. We estimated the transmissibility change of COVID-19 in China lately and obtained that the averaged basic reproduction number *R*_0_ of China COVID-19 was 3.44, 95% CI (2.87, 4.0). Up to now, the instantaneous basic reproduction numbers *R*_0*t*_ have two waves and reaching peaks on Jan. 8 and Jan. 27, respectively. After reaching a peak on Jan. 27, *R*_0*t*_ showed a continuous decline trend. On Feb. 9, *R*_0*t*_ has fallen to 1.68, but it is still larger than 1.

We find that one unit increase in cumulative BDI may result in a decrease of 0.0295% in *R*_0*t*_. The intensity of national prevention and control propaganda has continuously strengthened, and people can obtain more related information of COVID-19 on the Internet, such as disease situation report, prevention and control measures, and personal protection measures. With the increase of cumulative information, the public health awareness increases gradually, which may be conducive to implement prevention and control policies. Furthermore, we find that the effect of the Wuhan lockdown strategy plays an important role in decreasing the transmissibility of COVID-19. The instantaneous basic reproduction numbers *R*_0*t*_ reach the maximum on Jan. 27, 2020, and then a sustained decline (see Figure 1(a)). The regression analyses quantitatively find that one unit of BMS decrease may reduce the transmissibility change of COVID-19 by 2.7% (95% CI: 0.382, 4.97).

The current prevention and control measures have effectively reduced the transmissibility of COVID-19; however, the instantaneous basic reproduction number *R*_0*t*_ is still larger than the threshold 1; therefore, to minimize the risk of spreading the infection, ongoing travel restriction and public health awareness remain essential to provide a foundation for controlling the outbreak of COVID-19.

## Figures and Tables

**Figure 3:**
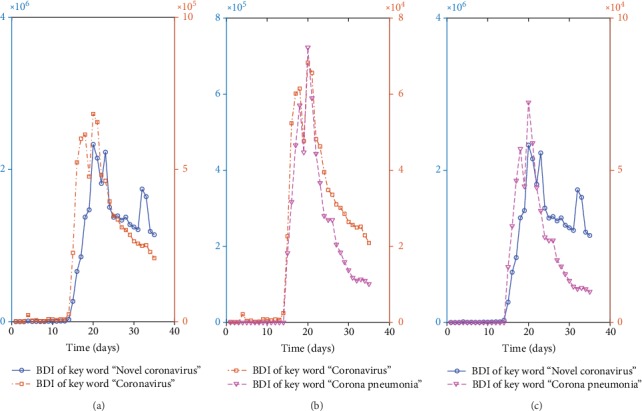
The BDI of keywords “novel coronavirus” (blue line), “coronavirus” (brown line), and “coronavirus pneumonia” (pink line) in China from Jan.6, 2020, to Feb. 9, 2020. (a) BDI of “novel coronavirus” and “coronavirus,” (b) BDI of “coronavirus” and “coronavirus pneumonia,” (c) BDI of “novel coronavirus” and “coronavirus pneumonia”.

**Table 1:** The summary result of transmissibility change of COVID-19 and its potential factors.

SI mean	SI SD	Averaged *R*_0*t*_ (95% CI)	*R*_0*t*_ vs. Cum. Baidu Index			*R*_0*t*_ vs. Baidu Migration Scale Index		
			Changing percentage (95%)	R-squared, *P* value	GCT, *P* value	Changing percentage (95%)	R-squared, *P* value	GCT, *P* value
7.5	3.4	3.44 (2.87, 4)	-0.029%^∗^ (-0.007%,-0.051%)	0.2845, *P* = 0.011	*P* < 0.05	-2.7%^∗^ (-4.97%, -0.38%)	0.137, *P* = 0.024	*P* < 0.05

^∗^The significant level is 0.05. *R*_0*t*_ is the instantaneous reproduction number, and SI is the serial interval of COVID-19; the changing percentage indicates that the change of *R*_0*t*_ when the predictors (e.g., Cum. Baidu Index or Baidu Migration Scale Index) increases one unit. GCT is the Granger causality test. Cum. Baidu Index is the Cumulative Baidu Index.

**Table 2:** The correlation coefficients of the three BDIs.

Pearson's correlation coefficients Spearman's rank correlation coefficients	BDI of “novel coronavirus”	BDI of “coronavirus”	BDI of “coronavirus pneumonia”
BDI of “novel coronavirus”	1	0.875^∗∗^ (*P* < 0.001)	0.84^∗∗^ (*P* < 0.001)
		0.847^∗∗^ (*P* < 0.001)	0.785^∗∗^ (*P* < 0.001)
BDI of “coronavirus”	0.875^∗∗^ (*P* < 0.001)	1	0.96^∗∗^ (*P* < 0.001)
	0.847^∗∗^ (*P* < 0.001)		0.965^∗∗^ (*P* < 0.001)
BDI of “coronavirus pneumonia”	0.84^∗∗^ (*P* < 0.001)	0.96^∗∗^ (*P* < 0.001)	1
	0.785^∗∗^ (*P* < 0.001)	0.965^∗∗^ (*P* < 0.001)	

^∗∗^Correlation is significant at the 0.01 level (2-tailed). The first line of the result is Pearson's correlation coefficients and *P* value, and the second line is Spearman's rank correlation coefficients and *P* value.

## Data Availability

The data used to support the findings of this study is available from the online website or corresponding author upon request.

## Appendix

### A. Baidu Index and Baidu Migration Scale

#### A.1. Baidu Index

Baidu Index [14] (BDI) is based on the search volume of the netizen in Baidu as the database, and keywords are used as statistical objects. The weighted sum of the search frequency of each keyword in Baidu web search was calculated as BDI. The BDI can reflect the degree of attention of internet users and also represent the constantly changing of hot spots. According to the different data sources, the Baidu Index can be divided into the PC Baidu Index and mobile Baidu Index. In our work, we used the sum of the PC Baidu Index and mobile Baidu Index.

The brief algorithm of the Baidu Index is as follows:

Step 1: Recorded the search volume of webpage based on the keywords and further carried out the filters and adjusted the weight.

Step 2: According to the search volume, the system can calculate the BDI. The BDI is a moving average, which can be calculate by the ratio of the search volume of webpage based on the keywords in comparative period to that volume of baseline period based on the same keywords. That is, the data in comparative period is obtained by comparing the intraday search volume of the users with the volume of related Baidu news in the past 30 days.

#### A.2. Baidu Migration Scale

The Baidu Migration Scale (BMS) [15] reflects the size of the population moving in or out one place in China. The Baidu Corporation carried out the full-sampled data processing on the positioning big data, explored the population migration changes, and visualized the population migration trajectory according to the changes in the positioning data of hundreds of millions of mobile phones.

The Baidu Migration Scale was calculated and analyzed based on the Baidu location-based service (LBS) open platform. By mining and analyzing the data of the Baidu map LBS development platform responding to 10 billion positioning requests every day, the trajectory and characteristics of the population migration in China were fully, dynamically, instantly, and intuitively displayed [19].

### B. The Selection of the Keywords of the Baidu Index

In our work, we used the Baidu Index data from China's largest search engine. The China people speak and write in Chinese, so we, respectively, collected the BDI data according to the Chinese terms “novel coronavirus,” “coronavirus,” and “coronavirus pneumonia,” and all other settings are the same (see Figure 3 and Table 2 for more details).

By comparing the three different keywords of COVID-19, we aim to explain why we chose the keyword “novel coronavirus” in our main context. From Figure 3, we can observe that these three time series of BDI have similar trends and inflection points (or peak). In addition, Pearson's correlation and Spearman's rank correlation coefficients among these three time series are estimated in Table 2. These results indicate that the Baidu Index of “novel coronavirus” can be selected to measure the public awareness with respect to COVID-19. Thus, the results based on BDI of “novel coronavirus” is consistent with those result based on the other two BDIs.
